# Long-term treatment with low dose glucocorticoids in Rheumatoid Arthritis: New tricks of an old drug

**DOI:** 10.31138/mjr.29.1.13

**Published:** 2018-03-19

**Authors:** Spyros N. Nikas

**Affiliations:** Private Practice Rheumatologist, Ioannina, Greece

**Keywords:** Glucocorticoids, rheumatoid arthritis

## Abstract

Glucocorticoids (GC) have been used for more than 70 years in the management of rheumatoid arthritis (RA). The immediate improvement of the clinical picture is one of their main advantages. However, RA is a chronic disease and unfortunately, long-term GC administration is associated with several serious adverse events. This is the major reason why GC therapy should be administered for the shortest possible period of time, with tapering as far as it is feasible. Although this is accepted as a “dogma”, there is recently growing evidence suggesting that low doses, even in the long-term, might not be as dangerous as previously believed. On the contrary, GC may be beneficial for RA patients in several ways, considering their protective role in radiographic progression or lymphoma development.

The beneficial effects of Glucocorticoids (GC) in patients with Rheumatic Diseases have been described for more than a half a century. It was 1949 when Philip S. Hench first administered daily intramuscular injections of 100mg 17-hydroxy-11-dehydrocorticosterone (compound E) in a 29-year-old, nearly bedridden patient with severe rheumatoid arthritis (RA). A dramatic clinical effect was seen within a week, with resolution of articular and muscular stiffness.^1^ Subsequently, the same regimen of adrenocorticotropic hormone (ACTH) was tried in other RA patients, with the same clinical response. For this milestone observation, Hench was honored with the Nobel Prize in Medicine in 1950. A few years later, other studies followed, comparing cortisone with acetyl salicylic acid^2^ or prednisolone with non-steroidal anti-inflammatory drugs (NSAIDs) and aspirin.^3^ The latter was actually the first study that demonstrated that GC, beyond their dramatic clinical efficacy in RA patients, also bear disease-modifying anti-rheumatic drug (DMARD) properties, i.e. that they inhibit radiological progression. It was also noticed that the long-term GC optimal daily dose should not exceed 10mg.

Recent understanding of pathophysiology suggests that inflammatory disorders might indeed “need” steroids. It is well-known and frequently described that these diseases, including RA, are in a kind of “glucocorticoid-deficient state”, due to several reasons, such as hereditary GC resistance due to abnormalities of the GC receptor, or inadequate secretion of GC in relation to inflammation.^4^ It is therefore important to continuously try to re-evaluate the therapeutic role of GCs in RA and other inflammatory conditions, based on the latest evidence.

The therapeutic role of GC in RA management has been described in the latest EULAR recommendations, where there is a suggestion of short-term use when initiating or changing conventional synthetic (cs)DMARDs, tapering a as soon as clinically feasible.^5^ Compared to the previous EULAR recommendations of 2013,^6^ a slight difference in emphasis is noticeable, in that the 6-month limit of usage and the exact dose recommendations have been removed. It is widely accepted that low doses of GC co-administration in RA treatment (≤ 7,5 mg prednisone per day) are being considered as “bridging therapy”; eliminating articular symptoms, from the day that csDMARDs are introduced until the day they start to provide benefit. It is quite interesting that GC are not recommended in patients initiating biologic (b)DMARDs or targeted synthetic (ts)DMARDs, mainly because these drugs are often characterized by a rapid onset of clinical action. Moreover, several recent studies have shown increasing risk of morbidity in patients with RA treated with a combination of GC and bDMARDs.^7^

The important role of GC in RA management is also highlighted by a few other recommendations: all of them indicate that GC, especially at low doses and for a short duration, remain an appropriate option in RA treatment.^8^ Unfortunately, most of those recommendations are based on data extracted from observational and retrospective studies, with a possible selection bias. The main reason must be that GC are old, cheap drugs, and therefore no company would consider spending the necessary cost to fund this much-needed research.^9^ However, new data concerning GC safety came from the recently published ESPOIR cohort study, where RA patients with low-dose GC treatment displayed, in a 7-year analysis, approximately the same safety profile as patients without GC.^10^ As it was expected, patents on GC (n=368) had more aggressive disease compared to patents without GC (greater use of NSAIDs, csDMARDs and bDMARDs, more active disease and more disability), and showed numerically a higher number of total adverse events (n= 44 vs 21) and infections (n=16 vs 3), compared to patients without GC exposure; the difference was not statistically significant (p=0.520 and p=0.09 respectively). In a further weighted Cox proportional-hazards analysis, composite outcomes do not differ with and without GC (p=0.520; HR=0.889; 95% CI 0.620 to 1.273). ESPOIR was the first study specifically designed to address GC adverse events in patients with RA. Despite these very encouraging data, the association between infections and GC therapy remains a controversial issue. There are several observational retrospective studies, showing that the frequency and severity of infections is similar between RA patients receiving low-dose GC with those who do not.^11,12,13^ On the other hand, there are studies where current or prior oral GC therapy, even at low doses, is associated with increased infection risk, mainly in older patients or in those with comorbidities.^14,15,16^

However, which are the most frequent GC adverse events? On a basis of patients’ self-reported information, Pincus et al. reported that bruising and skin-thinning, followed by low levels of hypertension, diabetes, and cataracts, were the primary adverse events, noting that doses of <5 mg/day over long periods appear acceptable and effective for many patients with RA.^17^ Weight gain (in one study occurring in up to 70% of patients) and hypertension were reported as the most common self-reported GC adverse events in other studies.^18,19^ From a patient’s point of view, sleep disturbance and weight gain seem to be the most undesirable adverse events.^8^

EULAR’s first approach on how to monitor low dose GC adverse events, back in 2010, specifically emphasized that standard care monitoring does not need to be extended for patients on low-dose GC therapy, except specifically for osteoporosis.^20^ However, the most interesting information arises from the latest EULAR viewpoints, where there is an agreement that the risk of harm is low for the majority of patients on long-term dosages of ≤5 mg prednisone equivalent per day (with the exception of cardiovascular disease [CVD]).^21^ At dosages of >10 mg/day, the risk of harm is elevated, whereas at dosages between >5 and ≤10 mg/day, patient-specific characteristics determine the risk of harm. Osteoporosis, hyperglycaemia, CVD and infections were considered the four most worrisome adverse effects of GC. RA is characterized by increased CVD risk and GC therapy may exhibit a dual effect. By decreasing inflammation, GC administration may be associated with decreased CV complications, since inflammation is a major contributor to the progression of atherosclerosis and plaque rupture. On the other hand, chronic and cumulative GC administration is characterized by proatherogenic lipid profiles, hypertension, insulin resistance, atherosclerosis and acute coronary syndromes.^22^ Regarding osteoporosis, very recently, ACR published the latest guidelines for the prevention and treatment of glucocorticoid-induced osteoporosis, concerning patients beginning or continuing long-term (≥ 3months) GC treatment.^23^ Physicians must always bear in mind that even short-term, low-dose oral GC therapy may be associated with a low, but statistically significant, increased incidence (RR: 2.763, P<.001) of osteonecrosis.^24^ On the other hand, the latest data about GC and gastrointestinal bleeding seems to be encouraging, since the increased risk is statistically significant only for hospitalised patients (OR 1.42, 95% CI 1.22 to 1.66).^25^

One of the long-term advantages of low-dose GC administration, besides the well-known excellent clinical response or safety, is a “money-saving” role; implying the reduction of potential future biologic exposure. Safy et al. have recently shown that the addition of 10 mg prednisone daily to a methotrexate-based treatment strategy in early PA resulted in a lower initiation rate of a first bDMARD.^26^ Moreover, they showed that this strategy, without increased GC-related comorbidities, was associated with significantly better radiographic outcomes, which actually raise the question, “Are GC really DMARDs?”

In a relevant paper, Boers et al. assess the damage progression in patients who were randomized to placebo in 3 placebo-biologic arms of RA biologics trials.^27^ Although this sub-analysis refers to patients who had already failed to respond to a first csDMARD, those on GC had reduced 6-month radiographic progression (in the pooled infliximab studies, by 2.6 points [95% CI 0.6–4.5]) compared to those without GC exposure. Even though there are studies with different conclusions,^28^ beneficial GC effects on structural damage are supported by several other studies.^29,30,31,32^ Even more, interesting findings arise from the meta-analysis by Graudal et al., where differences in clinical and radiographic outcomes between patients in combination treatment (triple therapy) or tumor necrosis factor inhibitors plus methotrexate were not present in cases of GC co-administration.^33^ A few years ago, a similar meta-analysis of 70 randomized placebo-controlled or drug-controlled studies revealed that direct comparison between the combination of a biologic agent plus MTX and the combination of 2 csDMARDs plus initial glucocorticoids showed no difference in radiographic progression.^34^ A definitive answer to the role of GC on radiographic progression is probably given by Kirwan’s Cochrane Database Systematic Review, where the structural beneficial effect of GC was mainly established in RA patients with early disease or in combination with csDMARD (standardised mean difference in progression was 0.40 in favour of glucocorticoids [95% CI 0.27, 0.54]).^35^ It is quite interesting that even before all these encouraging data were published, many experts tended to recommend low-dose GC co-administration actually in every patient with RA.^36^

It also seems that there is another reason for this, beyond the direct clinical efficacy (bridging therapy) or inhibition of radiologic progression: very recently, Hellgren et al. have shown that the addition of GC as a part of initial RA treatment (within 1 year) was associated with a reduced risk (HR 0.5 [95% CI 0.3–0.9]) of lymphoma development.^37^

In conclusion, there is no doubt that long-term GC administration associates with toxicity, dependent mainly on dose, duration, patient age and comorbidities. This is why the recommendation of “the minimal dose, for the minimal duration” still stands. On the other hand, it seems that fears about adverse events associated with higher doses have been expanded to the lower doses as well. Although there are earlier opposing data, mainly in the issue of infections, based on recent findings, long-term low dose GC administration seems to be not as harmful as previously thought, and may, in contrast, provide significant benefits, including rapid clinical improvement, disease-modifying inhibition of structural progression, reduction of lymphoma incidence and even cost savings.
